# Comparison of patient-controlled epidural analgesia and patient-controlled intravenous analgesia after spinal fusion surgery: a meta-analysis of randomized controlled trials

**DOI:** 10.1186/s12891-015-0849-y

**Published:** 2015-12-15

**Authors:** Peng Tian, Xin Fu, Zhi-jun Li, Xin-long Ma

**Affiliations:** Department of Orthopedics, Tianjin Hospital, No. 406, Jiefang Nan Road, Tianjin, 300211 People’s Republic of China; Department of Orthopedics, General Hospital of Tianjin Medical University, No.154, Anshan Road, Tianjin, 300052 People’s Republic of China

**Keywords:** Epidural analgesia, Intravenous analgesia, Spinal fusion, Randomized controlled trial, Meta-analysis

## Abstract

**Background:**

The objective of this meta-analysis was to compare the efficacy and safety of patient-controlled epidural analgesia (PCEA) and patient-controlled intravenous analgesia (PCIA) in postoperative analgesia of spinal fusion surgery.

**Methods:**

Potential academic articles were identified from the Cochrane Library, Medline (1966–2015.5), PubMed (1966–2015.5), Embase (1980–2015.5) and ScienceDirect (1966–2015.5). Gray studies were identified from the references of the included literature. Randomized controlled trials (RCTs) involving PCEA and PCIA after spinal fusion were included. Two independent reviewers performed independent data abstraction. I^2^ statistic was used to assess heterogeneity. Fixed or random effects model was used for meta-analysis.

**Results:**

Eight RCTs met the inclusion criteria. There was a better analgesic effect in patients with PCEA for postoperative VAS on the first day (*P* = 0.0005) and second day (*P* = 0.006). The patients with PCEA had a higher incidence of pruritus (*P* = 0.02) and paresthesia (*P* = 0.03) after surgery than those with PCIA. There was no statistically significant difference in postoperative VAS on the third day (*P* = 0.15), nausea (*P* = 0.74) or emesis (*P* = 0.37) between the two groups.

**Conclusions:**

After spinal fusion, the patients with PCEA have similar analgesic efficacy during the three postoperative days and a higher incidence of pruritus and paresthesia than those with PCIA. Due to the limited quality and data of the evidence currently available, more high-quality randomized controlled trials are required.

## Background

Low back pain (LBP) is an increasing clinical complaint in modern society; the condition affects up to 80 % of the population during their lifetime [1]. LBP is known to be associated with degenerative spinal diseases [2] such as lumbar disc herniation [3], lumbar spondylolisthesis [4], and adolescent idiopathic scoliosis [5]. Spinal fusion has been applied in the treatment of various degenerative spinal diseases [6]. Furthermore, this trend is being further enhanced owing to the development of surgical techniques and spinal instrumentation. Spinal fusion aims to alleviate pain by eliminating the painful spinal motion segments. Many patients who receive successful spinal fusion surgery obtain good outcomes [7–9].

However, major spinal surgeries involving spinal fusion cause severe postoperative pain, which most often lasts for the first three postoperative days [10, 11]. Because postoperative pain control is difficult and complex, it is an important aspect of patient care after spinal fusion [12, 13]. Apfelbaum et al. [14] reported that more than 70 % of patients experienced moderate to severe postoperative pain, and almost 25 % of patients had adverse effects with postoperative analgesia. Effective postoperative analgesia could not only improve patients’ satisfaction, but also reduce the incidence of postoperative complications and shorten the length of hospitalization [15, 16]. A multimodal approach is used for pain management, including patient-controlled intravenous analgesia (PCIA), patient-controlled epidural analgesia (PCEA), opioid medication, benzodiazepines, and oral acetaminophen.

PCEA plays a direct role in the near operative region, so the analgesic effect of PCEA is clear and fast. PCIA acts on a systemic level via intravenous analgesia drugs, so the analgesic effect of PCIA lasts substantially longer. Although both forms of analgesia are often used in spinal fusion, controversies over their efficacy and safety still exist. For patients who undergo spinal fusion, there are no systematic studies or reviews determining the difference between PCEA and PCIA. The purpose of the present meta-analysis is to compare the efficacy and safety of PCEA versus PCIA in patients with spinal fusion surgery from randomized controlled trials (RCTs).

## Methods

### Inclusion and exclusion criteria

RCTs were included if the following criteria were met: (1) Study design: RCT; (2) Study object: patients with spinal fusion surgery; (3) Postoperative analgesic interventions: Patients in the PCEA group received PCEA; analgesic drugs could include the single use of a local anesthetic drug or the combined use of opioids. Patients in the PCIA group received PCIA; analgesic drugs could include the single use of a local anesthetic drug or the combined use of opioids. Analgesic drugs may include the use of opioids or combined use of other drugs; (4) Outcome measures: The VAS scores of patients with spinal fusion on the first, second and third postoperative days during postoperative analgesia; Adverse effects including nausea, vomiting, skin itch, and paresthesia. RCTs were excluded if (1) The combined application of other measures besides analgesic interventions could have an impact on the final analysis; (2) the patients received spinal nonfusion; (3) the literature contained no associated data.

### Search strategy

PubMed, Medline, EMBase, Cochrane library, Science Direct and Web of Science were searched in March 2015 for RCTs involving PCEA and PCIA in the management of pain relief after spinal fusion. The search terms were as follows: epidural analgesia; intravenous analgesia; spinal fusion. No restrictions were imposed on language. The reference lists of all the eligible studies and relevant reviews were examined to identify any initially omitted studies. The included studies were published in a peer-reviewed journal as a full article, excluding the gray literature and conference proceedings.

### Quality assessment and data extraction

Two reviewers independently evaluated the bias risk of RCTs included in the study according to the RCT bias risk assessment tools of the Cochrane Handbook Version 5.1. For each eligible study, both reviewers extracted all the relevant data independently. Any disagreement was resolved by discussion; when no consensus could be achieved, a third reviewer acted as the adjudicator and made the final decision. Whenever necessary, reviewers contacted the authors of the studies for missing data or further information. The following data were extracted: (1) demographic data of participants; (2) indications for spinal fusion; (3) analgesic drugs, position of the catheter, analgesic efficacy evaluation index, and the incidence of adverse reaction after analgesia; (4) any other outcomes as mentioned in individual studies were considered for inclusion. In studies in which data were incomplete or unclear, attempts were made to contact the investigators for clarification.

### Data analysis and statistical methods

The meta-analysis was conducted with Review Manager software 5.2 for Windows (RevMan Version 5.2; The Nordic Cochrane Center, The Cochrane Collaboration, Copenhagen, Denmark). Statistical heterogeneity was assessed for each study, using a standard Chi square test, with significance set at a P value of 0.1, which was measured by the I^2^ statistic. When I^2^ > 50 %, *P* < 0.1 was considered to be significant heterogeneity. Therefore, a random-effects model was applied for data analysis. A fixed-effects model was used when no significant heterogeneity was found. In cases of significant heterogeneity, subgroup analysis was performed to investigate sources. For continuous outcomes, mean differences (MDs) and 95 % confidence intervals (CIs) were presented. Relative risk (RR) and 95 % CIs were calculated for dichotomous data.

## Results

### Literature search

**Fig. 1:**
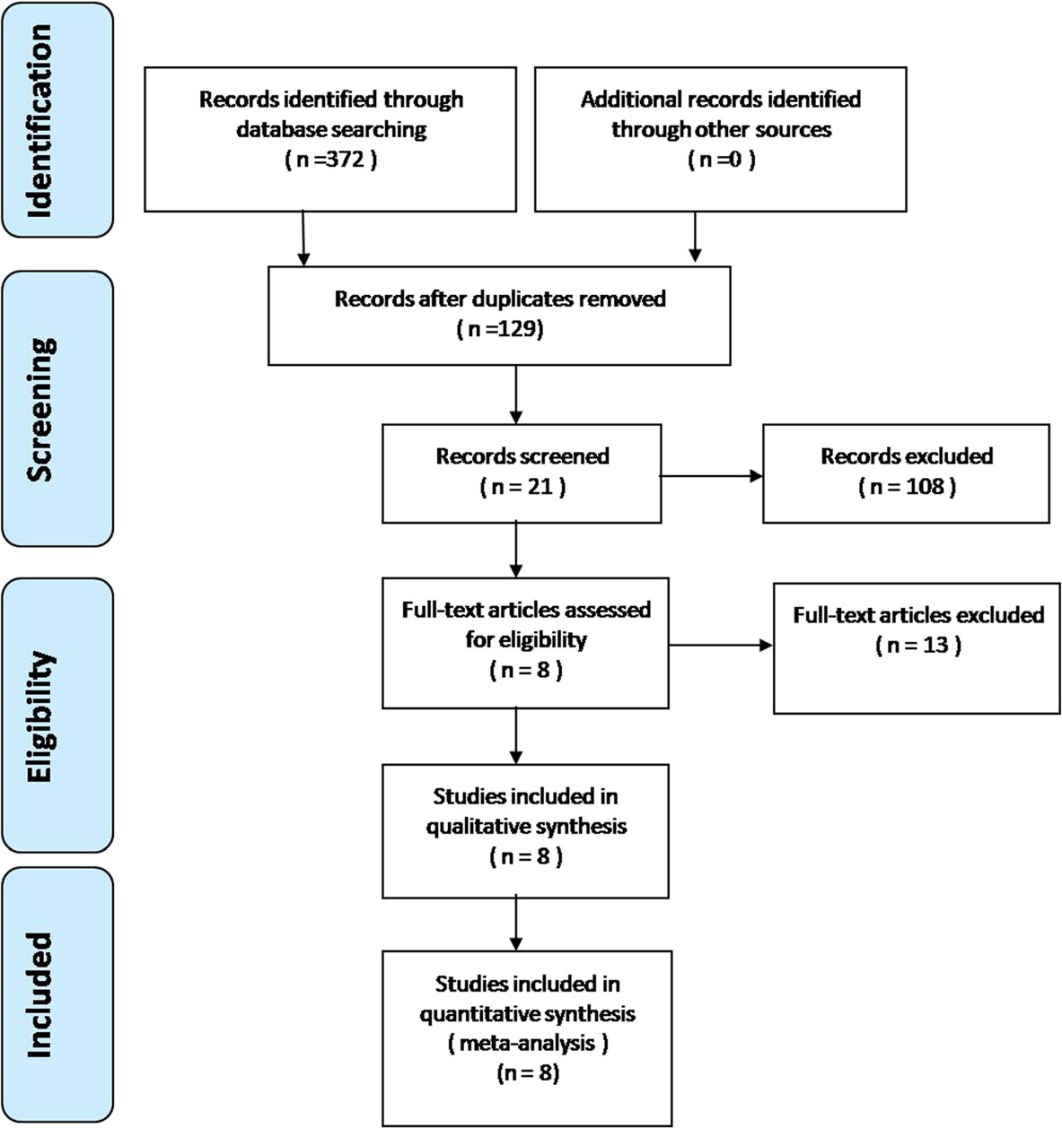
Flowchart of the study selection process

A total of 372 potential studies were identified using the first search strategy. 67 reports were excluded during screening of titles and 41 reports were excluded after screening of abstracts. Finally, 364 reports were excluded according to the eligibility criteria. No additional studies were obtained after the reference review. After careful full-text evaluation, eight independent RCTs [17–24] with 482 patients were included in the current meta-analysis as indicated by the flowchart in Fig. 1.

### Study characteristics

The main characteristics of the included studies are reported in Table 1. The sample size of included studies ranged from 33 to 74 patients. Statistically similar baseline characteristics were observed between PCEA and PCIA groups, including age, sex ratio and so on. Drug and dosage varied among studies.

**Table 1:** The main characteristics of included studies

Disease		Patient-controlled epidural analgesia (PCEA)				Patient-controlled intravenous analgesia (PCIA)		
		N(M/F)	Average age	Drugs	POC	N(M/F)	Average age	Drugs
Joshua 2013 [18]	AIS	44	NM	Bupivacaine Fentanyl	5 cm up to the middle of fused centrum	22	NM	Morphine
Kluba 2010 [19]	IDD	29 (14/15)	57	Ropivacaine Sufentanil	3 cm up to the middle of fused centrum	23 (14/9)	62	Piritramid
Gauger 2009 [17]	AIS	19 (0/19)	15.1	Bupivacaine Hydromorphone	3–5 cm up to middle of fused centrum	19 (4/15)	14.7	Hydromorphone
Schenk 2006 [23]	LS	28 (15/13)	42	Ropivacaine Sufentanil	At the middle of fused centrum	30 (21/9)	50	Morphine
Fisher 2003 [22]	IDD	36 (17/19)	50	Fentanyl Bupivacaine Epivacaine	10 cm up to the middle of fused centrum	38 (15/23)	51	Fentanyl
Cassady 2000 [20]	AIS	17 (2/15)	14.6	Bupivacaine Epivacaine	NM	16 (3/13)	14.4	Morphine sulfate
Coben 1997 [21]	IDD	21	45	Morphine sulfate	NM	21	45	Morphine sulfate Bupivacaine
Johnson 1989 [24]	IDD	29 (21/8)	39	Fentanil Morphine sulfate	2–3 cm up to middle of fused centrum	13 (8/5)	39	Morphine sulfate

N*(M/F)* numbers(male/female), *POC* position of catheter, *AIS* adolescent idiopathic scoliosis, *IDD* intervertebral disc degeneration, *LS* lumbar spodylolithesis

### Risk of bias assessment

**Fig. 2:**
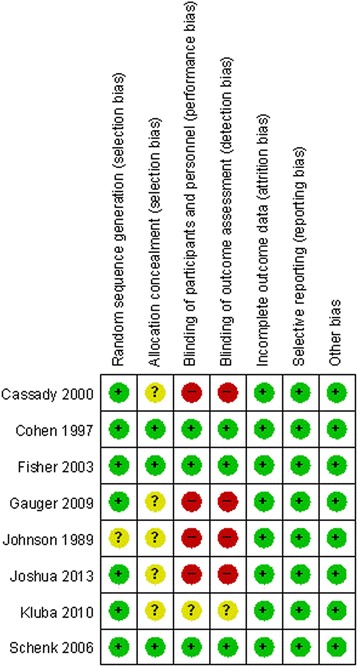
The summary of bias risk of included studies

The quality of the included studies according to the Cochrane Handbook for Systematic Review of Interventions is reported in Fig. 2. Randomization was not clear for one RCT [20]. Adequate concealment of allocation was clear for three studies [21–23]. Three included studies [21–23] stated blinding.

### Outcomes for meta-analysis

#### Postoperative visual analogue scale (VAS) scores

A VAS with a range from 0 to 10 was used to assess subjective pain intensity. The VAS score was used to evaluate the postoperative pain condition of patients who received PCEA and PCIA in all included studies. VAS scores on the first, second and third postoperative days were conducted to meta-analysis respectively.

#### VAS score on first postoperative day

**Fig. 3:**
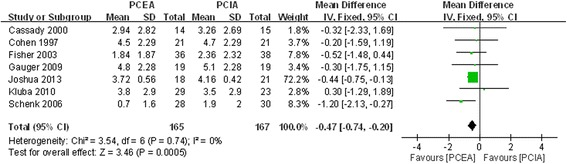
Forest plot of VAS score at post-operative first day between PCEA and PCIA

Seven included studies stated VAS score on the first postoperative day [17–23]. There was no significant heterogeneity (χ^2^ = 3.54, df = 6, I^2^ = 0 %, *P* =0.72; Fig. 3). The pooled results indicated that the analgesic effect of PCEA was better than that of PCIA; there was a statistically significant difference between the two groups (MD = −0.47, 95 % CI:−0.74 to−0.20, *P* =0.0005).

#### VAS score on second postoperative day

**Fig. 4:**
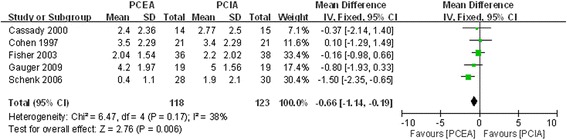
Forest plot of VAS score at post-operative second day between PCEA and PCIA

Five studies reported VAS score on the second postoperative day [17, 20–23]. There was no significant heterogeneity (χ^2^ = 6.47, df = 4, I^2^ = 38 %, *P* =0.17; Fig. 4). The pooled results indicated that the analgesic effect of PCEA was better than that of PCIA; there was a statistically significant difference between the two groups (MD = −0.66, 95 % CI:−1.14 to−0.19, *P* =0.006).

#### VAS score on third postoperative day

**Fig. 5:**
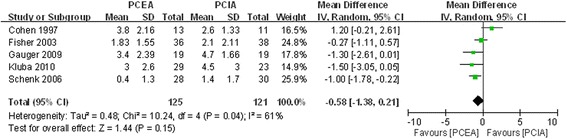
Forest plot of VAS score at post-operative third day between PCEA and PCIA

Five included studies assessed VAS score on the third postoperative day [17, 19, 21–23]. There was significant heterogeneity (χ^2^ = 10.24, df = 4, I^2^ = 61 %, *P* =0.04; Fig. 5). The pooled results indicated that the analgesic effect of PCEA was better than that of PCIA; there was no significant difference between the two groups (MD = −0.58, 95 % CI:−1.38 to 0.21, *P* =0.15).

### Adverse reactions

#### Nausea

**Fig. 6:**
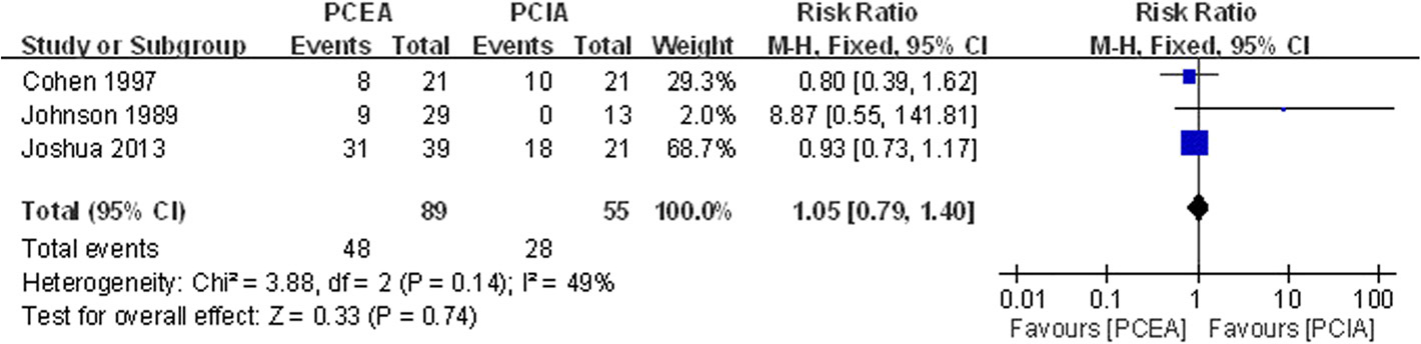
Forest plot of postoperative nausea between PCEA and PCIA

Three studies reported postoperative nausea after application of PCEA or PCIA [18, 21, 24]. There was no significant heterogeneity (χ^2^ = 3.88, df = 2, I^2^ = 49 %, *P* =0.14; Fig. 6). Pooled results demonstrated that the incidence of postoperative nausea showed no significant difference between the two groups (RR = 1.05, 95 % CI: 0.79 to 1.40, *P* =0.74).

#### Emesis

**Fig. 7:**
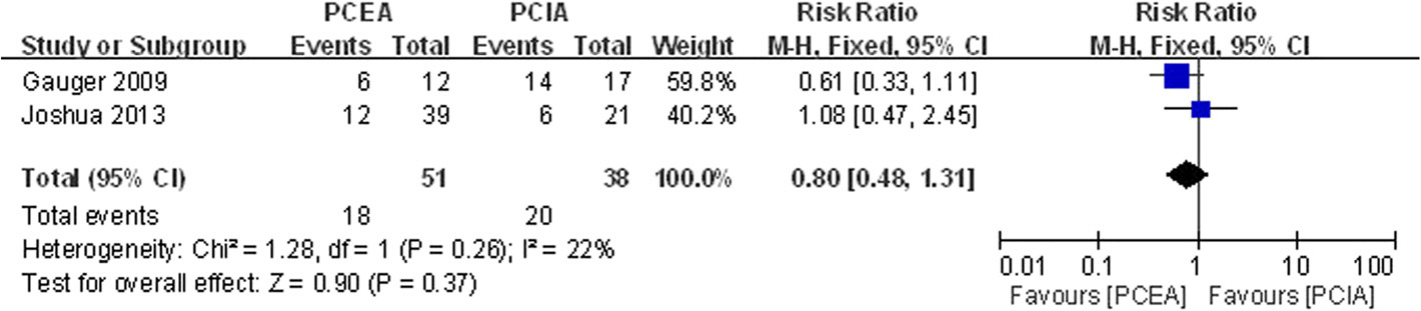
Forest plot of postoperative emesis between PCEA and PCIA

Two studies showed postoperative emesis after application of PCEA and PCIA [17, 18]. There was no significant heterogeneity (χ^2^ = 1.28, df = 1, I^2^ = 22 %, *P* =0.26; Fig. 7). Pooled results demonstrated that the incidence of postoperative emesis showed no significant difference between the two groups (RR = 0.80, 95 % CI: 0.48 to 1.31, *P* =0.37).

#### Pruritus

**Fig. 8:**
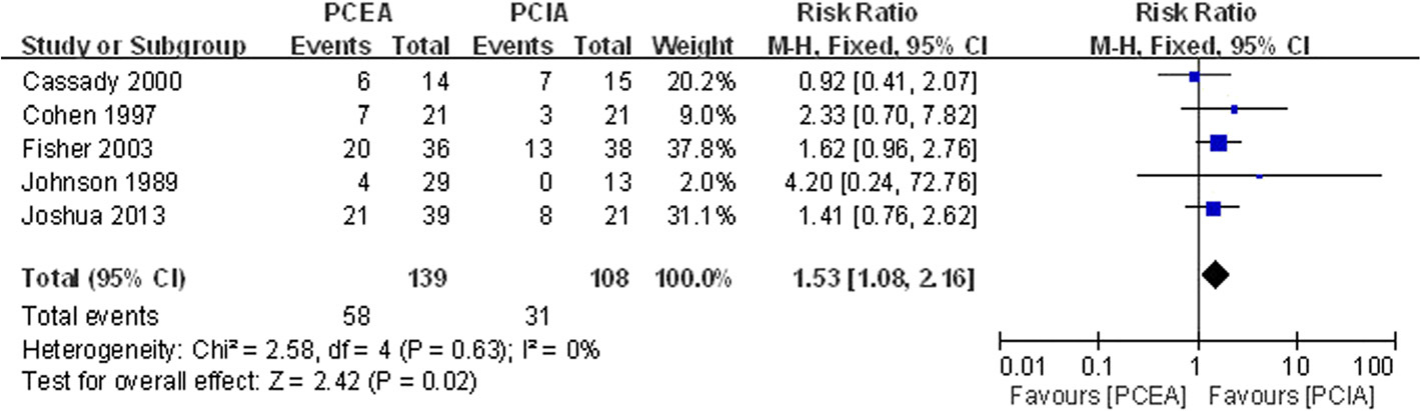
Forest plot of postoperative pruritus between PCEA and PCIA

Five studies documented postoperative pruritus after application of PCEA and PCIA [18, 20–22, 24]. There was no significant heterogeneity (χ^2^ = 2.58, df = 4, I^2^ = 0 %, *P* =0.63; Fig. 8). Pooled results demonstrated that the incidence of postoperative pruritus showed a significant difference between the two groups (RR = 1.53, 95 % CI: 1.08 to 2.61, *P* =0.02).

#### Paresthesias

**Fig. 9:**
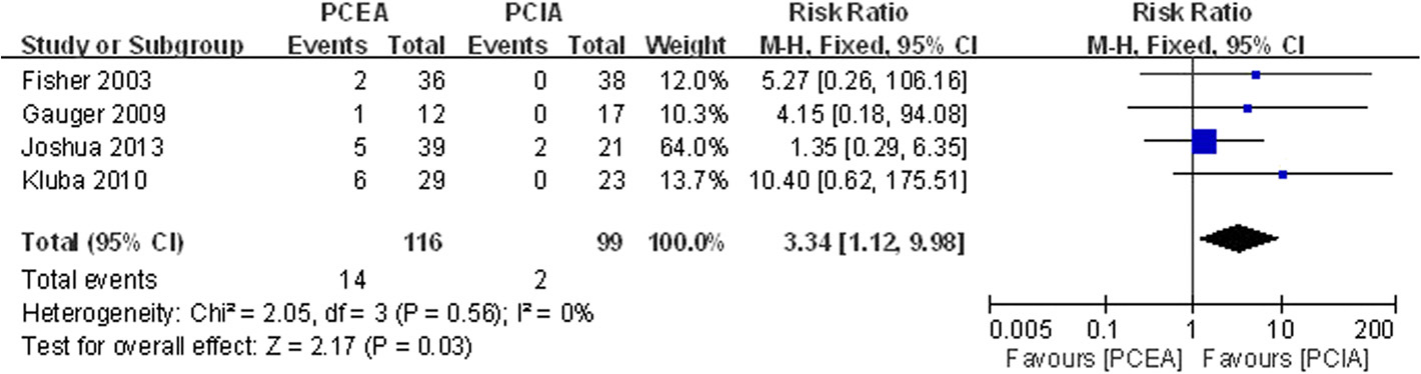
Forest plot of postoperative paresthesias between PCEA and PCIA

Four studies reported postoperative paresthesias after application of PCEA and PCIA [17–19, 22]. There was no significant heterogeneity (χ^2^ = 2.05, df = 3, I^2^ = 0 %, *P* =0.56; Fig. 9). Pooled results demonstrated that the incidence of postoperative paresthesias showed a significant difference between the two groups (RR = 3.34, 95 % CI: 1.12 to 9.98, *P* =0.03).

## Discussion

Patients’ postoperative satisfaction and functional recovery determine the overall efficacy of spinal fusion surgery. Effective analgesia can reduce or even eliminate pain so that patients achieve better results after rehabilitation. Therefore, it is imperative to choose the appropriate postoperative pain management strategy [25, 26]. The most important findings of the present meta-analysis are that the application of PCEA does not more effectively relieve in three postoperative day pain as compared to PCIA, meanwhile increasing the incidence of some complications such as pruritus and paresthesia.

Eight RCTs were reviewed in the current meta-analysis. Although the quality of the included studies was relatively high, there were still methodological weaknesses which should be taken into consideration. The blinding of four studies was not adequate; thus, detection bias may influence the results. Complete baseline data was shown in all literature. Due to the small sizes of the included studies, reviewers failed to conduct subgroup analysis to find the source of heterogeneity, though there was significant heterogeneity in some outcomes between groups.

Present meta-analysis showed that the analgesic effect on patients in the PCEA group was better than that in the PCIA group on the first and second postoperative days, but there was no significant difference in the analgesic effect on the two groups on the third postoperative day. Although there were significant differences in VAS score between two groups at the first and second postoperative day, the MD and 95 % CI were very small (MD = −0.47, 95 % CI:−0.74 to−0.20 and MD = −0.66, 95 % CI:−1.14 to−0.19 respectively). The extreme of the 95 % CI and this little MD were thus quite unlikely to be the actual difference between PCEA and PCIA. We should consider these when analysing the present findings. There was no difference in analgesic effect between PCEA and PCIA group at third postoperative day. There was no substantial difference of pain-relief between the PCEA and PCIA or at least that the demonstrated difference of pain relieve associated with two analgesic methods was so small that it could not be clinically relevant. These results were not consistent with some researches [27, 28]. In theory, PCEA seems to be an ideal method for postoperative pain control; it could be demonstrated to have a better postoperative analgesic effect in many common operations such as abdominal and gynecological surgeries [29, 30]. PCEA could possess a more immediate analgesic mechanism, thus reducing the use of opioids [21].

The diagnosis of patients received spinal fusion included scoliosis and other spinal degenerative diseases such as lumbar spondylolisthesis and lumbar spinal stenosis. The results demonstrated that there was a higher incidence of pruritus and paresthesia in patients with PCEA than those with PCIA (RR = 1.53, 95 % CI: 1.08 to 2.61, *P* =0.02 and RR = 3.34, 95 % CI: 1.12 to 9.98, *P* =0.03 respectively). But there was no significant difference in nausea and vomiting experienced between the PCEA and PCIA groups (RR = 1.05, 95 % CI: 0.79 to 1.40, *P* =0.74 and RR = 0.80, 95 % CI: 0.48 to 1.31, *P* =0.37 respectively). Nausea and vomiting are common side effects in the process of analgesic therapy [31]. Lumbar spinal stenosis could potentially increase the risk of patients with PCEA-induced paresthesia and motor palsy. Kluba study [19] and Fisher study [22] demonstrated that there was significantly higher incidence of paresthesia compared with the other studies. The use of postoperative PCEA should be cautious after the treatment of lumbar spinal stenosis and lumbar spondylolisthesis with spinal surgery. Only one study demonstrated that there were no significant differences between groups in VAS pain scores, side effects, or time to resumption of liquid intake between PCEA and PCIA [20], and the ages of patients in that study were lower than those of patients in other studies.

Ethnicity, age and genetics could influence the response to morphine; adverse reactions to analgesic drugs could be different [32]. Some reviews demonstrated that intravenous acetaminophen had no effect on gastrointestinal motility, renal function or bone healing [33]. In future research, subjects should be of similar age in order to reduce age bias.

It is imperative to acknowledge several potential limitations in our meta-analysis: (1) For the particularity of the clinical operation, the sample size of each trial was relatively small; (2) There were some differences in postoperative analgesia drugs and dosages; (3) There were some methodological weakness in all included RCTs. Because of the above defects and deficiencies, the pooled estimates should be explained with caution.

## Conclusion

After spinal fusion, the patients with PCEA have similar analgesic efficacy during the three postoperative days and a higher incidence of pruritus and paresthesia than those with PCIA. Due to the limited quality and data of the evidence currently available, more high-quality randomized controlled trials are required.

## Abbreviations

LBP: Low back pain
PCIA: Patient-controlled intravenous analgesia
PCEA: Patient-controlled epidural analgesia
RCTs: Randomized controlled trials
VAS: Visual analogue scale
MD: Mean difference
CI: Confidence intervals
RR: Relative risk
